# Relationship between nitrate headache and outcome in patients with acute stroke: results from the efficacy of nitric oxide in stroke (ENOS) trial

**DOI:** 10.1136/svn-2020-000498

**Published:** 2020-11-05

**Authors:** Lucy Beishon, Lisa J Woodhouse, Daniel Bereczki, Hanne K Christensen, Ronan Collins, John Gommans, Christina Kruuse, George Ntaios, Serefnur Ozturk, Stephen Phillips, Stuart Pocock, Szabolcs Szatmari, Joanna Wardlaw, Nikola Sprigg, Philip M Bath, ENOS Investigators

**Affiliations:** 1 Stroke Trials Unit, Division of Clinical Neuroscience, University of Nottingham, Nottingham, UK; 2 Department of Neurology, Semmelweis University, Budapest, Hungary; 3 Bispebjerg Hospital and University of Copenhagen, University of Copenhagen, Copenhagen, Denmark; 4 Department of Geriatric and Stroke Medicine, Adelaide and Meath Hospital, Dublin, Ireland; 5 Department of Medicine, Hawke's Bay Hospital, Camberley, New Zealand; 6 Department of Neurology, Herlev Gentofte Hospital, University of Copenhagen, Copenhagen, Denmark; 7 Department of Medicine, Larissa University Hospital, University of Thessaly, Volos, Greece; 8 Department of Neurology, Selcuk University Medical Faculty, Konya, Turkey; 9 Department of Neurology, Queen Elizabeth II Health Sciences Centre, Halifax, Nova Scotia, Canada; 10 Medical Statistics Unit, London School of Hygiene & Tropical Medicine, London, UK; 11 Department of Neurology, George Emil Palade University of Medicine, Pharmacy, Science and Technology of Târgu Mureș, Targu Mures, Romania; 12 Centre for Clinical Brain Sciences, University of Edinburgh, Edinburgh, UK; 13 Stroke, Nottingham University Hospitals NHS Trust, Nottingham, UK

**Keywords:** intervention, stroke, blood pressure

## Abstract

**Introduction:**

Nitrate-induced headache is common and may signify responsive cerebral vasculature. We assessed the relationship between nitrate headache and outcome in patients with acute stroke.

**Materials and methods:**

Patients were those randomised to glyceryl trinitrate (GTN) versus no GTN in the efficacy of nitric oxide in stroke trial. Development of headache by end of treatment (day 7), and functional outcome (modified Rankin Scale, primary outcome) at day 90, were assessed. Analyses are adjusted for baseline prognostic factors and give OR and mean difference (MD) with 95% CI.

**Results:**

In 4011 patients, headache was more common in GTN than control (360, 18.0% vs 170, 8.5%; p<0.001). Nitrate-related headache was associated with: younger age, female sex, higher diastolic blood pressure, non-total anterior circulation syndrome, milder stroke and absence of dysphasia (p<0.05). Nitrate headache was not associated with improved functional outcome (OR 0.90, 95% CI 0.73 to 1.10, p=0.30) or death (day 90) (HR 0.64, 95% CI 0.40 to 1.02, p=0.062), but reduced death or deterioration (day 7) (OR 0.45, 95% CI 0.25 to 0.82), death in hospital (OR 0.44, 95% CI 0.22 to 0.88) and improved activities of daily living (Barthel index, MD 3.7, 95% CI 0.3 to 7.1) and cognition (telephone interview cognitive screen, MD 2.0, 95% CI 0.7 to 3.3) (day 90). Non-nitrate headache was not associated with death, disability or cognition.

**Discussion and conclusion:**

Development of a nitrate headache by day 7 after stroke may be associated with improved activities of daily living and cognitive impairment at day 90, which was not seen with non-nitrate headache.

## Introduction

Headache is a common complication of stroke affecting ~25% of patients.^1^ Headache may be chronic, that is, preceding the stroke, or acute due to raised intracranial pressure, activation of nociceptive afferent nerves, altered cerebral perfusion and release of vasoactive substances.^2^ Headache may also be an adverse reaction of certain drugs, in particular those that modulate the nitric oxide-cyclic guanosine monophosphate (NO-cGMP) system such as nitrates (eg, glyceryl trinitrate, GTN) or phosdiesterase-5 inhibitors (PDE5-I, eg, dipyridamole) or the prostacyclin-cyclic adenosine monophosphate system such as phosdiesterase-3 inhibitors (eg, cilostazol).^3^ Trials have reported the rapid development of headache with GTN in patients with acute stroke (8%–18%),^4–8^ and dipyridamole (11%–38%)^9–12^ and cilostazol (23%)^13 14^ in chronic stroke. Nitrate-induced headache is thought to be mediated through changes in cerebrovascular tone, and the symptoms are comparable to those experienced in acute attacks of migrainous headache.^15 16^ Certainly, in migraine sufferers, nitrates can induce migraine headache, with increased intensity and greater dilation of cerebral vasculature.^15 16^ Thus, nitrate-headache may be a useful marker of intact vascular reactivity and treatment response. Although nitrate and PGI_2_-type headaches can be mitigated in part through weaning-up doses, the development of such headache makes compliance with these drugs a challenge, in both trials and routine clinical practice.

Headache at stroke onset has been reported previously to be associated with a poor outcome.^17^ In contrast, development of a dipyridamole-related headache was associated with reduced stroke recurrence and symptomatic intracranial haemorrhage (sICH) in patients with postischaemic stroke in the Prevention Regimen for Effectively Avoiding Second Strokes (PRoFESS) and the Second European Stroke Prevention Study (ESPS-2).^12^ This finding raises the hypothesis that headache occurring with other modulators of the NO-cGMP system might also improve outcome. However, no studies have specifically examined the relationship between nitrate headache, cerebral perfusion and outcome in acute stroke. Thus, the mechanisms by which nitrate headache may relate to improved outcomes in stroke remain unclear. Importantly, patients with nitrate headache may selectively benefit from nitrates through the maintenance of vascular reactivity. Therefore, the presence of headache may have prognostic significance in acute stroke and facilitate targeted intervention for these individuals.

The efficacy of nitric oxide in stroke (ENOS) trial randomised participants to GTN versus no GTN.^8^ The primary outcome of the trial (modified Rankin scale, mRS at day 90) was neutral.^8^ In this preplanned secondary analysis,^18^ we aimed to test the hypothesis that GTN-related headache is associated with an improved functional outcome.

## Methods

### Aims

We assessed the relationship between nitrate headache and outcomes at day 7 and 90 in patients with acute stroke. As a comparison, the same measures were assessed in those randomised to no GTN. Specifically, we explored the following:

1. Baseline demographics and neuroimaging features of participants with nitrate headache, and differences with non-nitrate headache.
2. Changes in blood pressure and haemodynamics in nitrate and non-nitrate headache.
3. The relationship between nitrate headache and clinical outcomes: function, death, death or deterioration, cognition, stroke recurrence, sICH, quality of life, cerebral oedema, infarct extension, mood and length of stay.

### ENOS trial

ENOS was an international randomised-controlled, patient-masked, blinded-endpoint trial; the protocol, statistical analysis plan, baseline characteristics and main results have been published previously.^8 18–20^ In brief, 4011 adult patients were recruited with acute stroke (ischaemic and haemorrhagic) within 48 hours of onset, with limb motor deficit and systolic blood pressure between 140 and 220 mm Hg. Participants were randomised (using stratification and minimisation) to receive transdermal GTN (5 mg) or no GTN (control) for 7 days. The trial had national research ethics committee and regulatory approvals in each country, and informed written consent was taken for each patient (or proxy consent from a relative if the patient lacked capacity).^19^ All baseline characteristics and other variables reported here, including the presence or absence of headache by day 7 (end of treatment) and outcomes at day 90, were collected prospectively. Headache was a patient reported outcome, occurring during GTN/no GTN treatment and necessitated intervention (analgesia), including withdrawing GTN/no GTN treatment. Headache was defined by the participant and the investigator. Nitrate headache was considered defined as a headache occurring in the context of nitrate treatment, and non-nitrate headache where this occurred in the no GTN arm.

The primary end point for the main trial analysis was functional outcome (mRS). Data were collected on the following secondary outcome measures: death (day 7 and 90, in hospital), death or deterioration (day 7), stroke recurrence (day 7), sICH (day 7), hypotension and hypertension (day 7), Scandinavian Stroke Scale (SSS, day 7), cerebral oedema (day 7), infarct extension (day 7), complication (day 7), length of stay, Barthel index (day 7), cognition at day 90 (Mini-Mental State Examination (MMSE), Modified Telephone Interview for Cognitive Status, category fluency), mood (Zung Depression Scale) and quality of life (European Quality of Life-5 dimensions (EQ-5D), European Quality of Life-Visual Analogue Scale). A full description and definition of the outcome measures has been published previously.^8 18 19^

### Statistical analysis

Nitrate headache was defined as a headache occurring in participants randomised to GTN; these headaches will have included some that were not related to nitrate treatment, as occurred in the non-GTN group. All analyses were performed by intention to treat. Baseline characteristics were compared within randomised groups between those with and without a headache by day 7 using χ^2^ test (binary variables), and independent t-test (continuous variables). The relationship between the development of headache and outcomes within treatment groups was assessed using regression models comprising binary logistic regression (death, death or deterioration, sICH, stroke recurrence by day 7), Cox proportional regression (death up to day 90), ordinal logistic regression (mRS at day 90^21^), and multiple linear regression analysis (other continuous variables). Regression analyses were performed unadjusted, and adjusted for^8^: age, sex, premorbid mRS, previous stroke, diabetes mellitus, current nitrate use, time from onset to randomisation, systolic blood pressure, total anterior circulation syndrome (TACS), SSS score, stroke type, thrombolytic treatment, feeding status, and continuation of prestroke antihypertensive drugs. The interactions between prespecified participant subgroups, headache and outcome (mRS) were assessed following adjustment using ordinal logistic regression with an interaction term. Time updated outcomes were produced for survival, where time in days was calculated from day-of-onset of headache to day-of-death and then categorised into dichotomous variables for those who died between days 0–7, 8–30, 31–90 or more than 90 days after headache onset. Each time updated outcome was entered into a binary logistic regression model and the effect of headache on survival at the different time points was analysed.

Results are presented as OR, HR or mean difference with 95% CIs. ORs represent the increase in risk of an outcome measure with a headache at day 7. Analyses were conducted with SPSS (V.22 for Mac or Windows), and significance was set at p<0.05.

## Results

Of the 4011 participants randomised into ENOS, 4000 had data recorded on the presence or not of headache. Of these, 530 had a headache by day 7; headache was more common in patients randomised to GTN (360, 18.0%) as compared with those randomised to no GTN (170, 8.5%) (p<0.001). Eleven participants did not have data reported on headache, therefore, the total number of participants included in this analysis was 4000. In participants randomised to GTN, those who developed a headache by day 7 were younger; more likely to be female; have higher diastolic blood pressure; more likely to have a mild stroke (higher baseline SSS score, more oral feeding), lacunar syndrome or ischaemic stroke; and less likely to have dysphasia or atrial fibrillation (table 1). In the control (no GTN) group, patients developing a headache were younger and were more likely to have neglect or hemianopia than those without headache. In respect of baseline imaging, nitrate-related headache was associated with less white matter change and lower white matter score; less atrophy; and fewer previous stroke lesions (table 1). Participants with a non-nitrate headache had fewer previous stroke lesions and a lower atrophy score on imaging than patients in the control group without headache.

**Table 1:** Baseline demographics by treatment group (GTN/no GTN), in patients with and without headache at day 7

	GTN	(n=1996)	P value	No GTN	(n=2004)	P value
Headache	Yes	No		Yes	No	
No of patients (n)	360 (18.0)	1636 (82.0)		170 (8.5)	1834 (91.5)	
Age (years)	66.6 (12.4)	71.1 (11.9)	<0.005	67.6 (11.8)	70.6 (12.2)	0.002
Sex, female (%)	172 (47.8)	679 (41.5)	0.029	82 (48.2)	777 (42.4)	0.14
Pre-morbid mRS score	0 (1)	0 (0)	0.006	0 (0)	0 (1)	0.12
Medical history (%)						
Hypertension	220 (61.1)	1065 (65.1)	0.15	105 (61.8)	1211 (66)	0.26
Stroke	51 (14.2)	262 (16)	0.38	22 (12.9)	255 (13.9)	0.73
Diabetes mellitus	52 (14.4)	289 (17.7)	0.14	29 (17.1)	324 (17.7)	0.84
Current nitrate use	18 (5)	69 (4.2)	0.51	6 (3.5)	61 (3.3)	0.89
Onset to randomisation (hours)	25.2 (12.5)	25.8 (12.9)	0.42	25.1 (12.5)	26.3 (12.9)	0.25
Haemodynamics						
Systolic BP (mm Hg)	166.7 (18.5)	167.5 (18.8)	0.46	168.4 (19.5)	167 (19.1)	0.36
Diastolic BP (mm Hg)	91.6 (12.7)	89.3 (13)	0.002	89.2 (12.5)	89.4 (13.4)	0.88
Heart rate, mean (bpm)	78.2 (14.8)	77.5 (14.8)	0.45	77.6 (14.3)	77.3 (14.7)	0.77
Atrial fibrillation (%)	53 (14.7)	336 (20.5)	0.012	33 (19.4)	338 (18.4)	0.75
Syndrome						
TACS (%)	86 (23.9)	527 (32.2)	0.002	58 (34.1)	535 (29.2)	0.18
LACS (%)	154 (42.8)	541 (33.1)	0.008	48 (28.2)	653 (35.6)	0.28
Dysphasia	90 (25.0)	704 (43.0)	<0.005	59 (34.7)	750 (40.9)	0.12
Neglect/inattention	91 (26.6)	431 (30)	0.21	61 (40.7)	485 (29.6)	0.005
Hemianopia	74 (20.6)	297 (18.2)	0.89	51 (30)	313 (17.1)	<0.005
SSS score (/58)	37.2 (11.2)	33.2 (13.4)	<0.005	33.8 (11.9)	33.5 (13.4)	0.70
Stroke type (%)						
Ischaemic	287 (79.7)	1373 (83.9)	0.043	129 (75.9)	1544 (84.2)	0.12
Haemorrhage	39 (10.8)	278 (17)		66 (38.8)	244 (13.3)	
Non stroke	2 (0.6)	11 (0.7)		7 (4.1)	19 (1)	
Lesion side (%)						
Left	109 (45.0)	501 (47)	0.78	49 (43)	541 (45.3)	0.50
Right	131 (54.1)	554 (51.9)		63 (55.3)	645 (54)	
Both	2 (0.8)	12 (1.1)		2 (1.8)	9 (0.8)	
Feeding status, non-oral (%)	76 (21.1)	591 (36.1)	<0.005	56 (32.9)	605 (33)	0.99
Neuroimaging adjudication						
White matter change (%)	116 (32.2)	701 (42.8)	<0.005	64 (37.6)	763 (41.6)	0.16
White matter score (/4)	0 (2)	0 (2)	<0.005	0 (2)	0 (2)	0.18
Atrophy (%)	272 (75.6)	1354 (82.8)	<0.005	135 (79.4)	1464 (79.8)	0.24
Atrophy score (/4)	2 (1)	2 (2)	<0.005	2 (1)	2 (2)	0.007
Previous stroke lesion (%)	194 (53.9)	992 (60.6)	0.006	81 (47.6)	1056 (57.6)	0.002
Alteplase	36 (10.1)	167 (10.2)	0.93	25 (14.9)	195 (10.7)	0.095
Continued antihypertensives (%)	81 (22.5)	432 (26.4)	0.009	38 (22.4)	486 (26.5)	0.26

Data are number (%), median (IQR) or mean (SD). Comparison by χ^2^, Kruskal-Wallis or independent t-test.

BP, blood pressure; GTN, glyceryl trinitrate; LACS, lacunar syndrome; mRS, modified Rankin Scale; SSS, Scandinavian Stroke Scale; TACS, total anterior circulation syndrome.

Of the patients who developed a headache, 13 (3.6%) in the GTN group and 2 (1.2%) in the non-GTN group had withdrawn from treatment by day 7 (p=0.12). There was no significant difference between the treatment groups for time from randomisation to development of the headache: GTN 1.2 (2.1) days vs no GTN 1.9 (6.9), mean difference −0.7 days (95% CI −1.8, 0.4 days, p=0.20). Changes in blood pressure and other haemodynamic measures between baseline and 1–2 hours postfirst dose (day 1) did not differ by the presence or absence of a headache in either treatment group (online supplemental table 1).

10.1136/svn-2020-000498.supp1 Supplementary data

**Figure 1:**
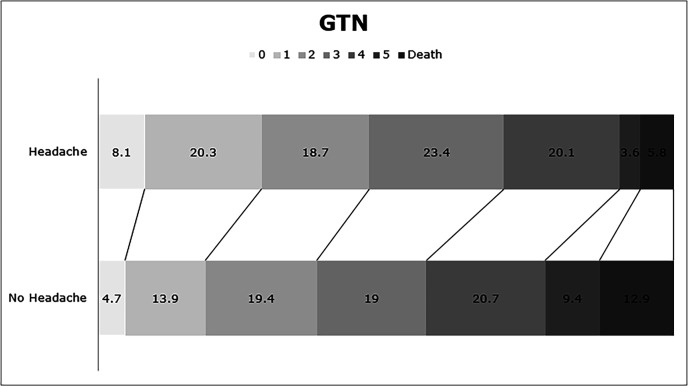
Distribution of modified Rankin Scale scores at day 90 by presence or absence of headache by day 7 for the GTN group. Data are percentage of total patients. Comparison by ordinal logistic regression. Adjusted OR 0.9 (95% CI 0.73 to 1.1), p=0.3 (no headache: n=1631, headache: n=359). GTN, glyceryl trinitrate.

**Figure 2:**
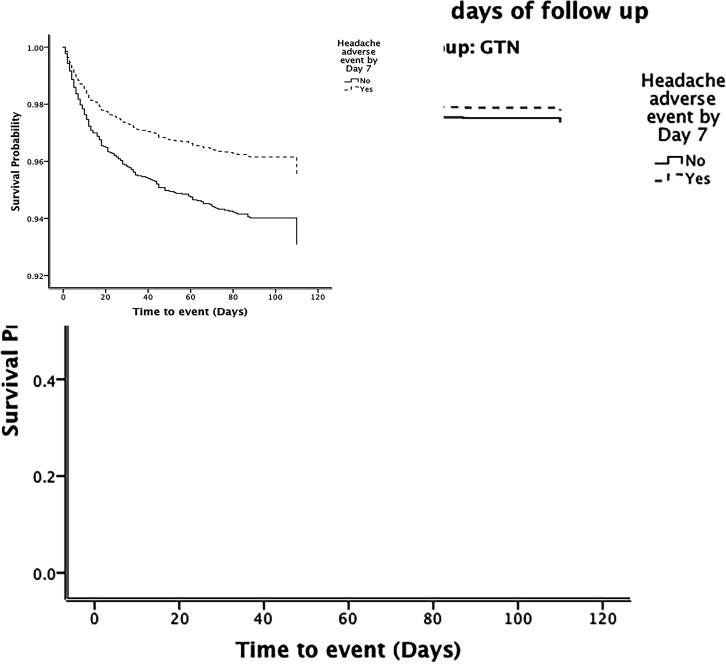
Survival curves over 90 days of follow-up for the glyceryl trinitrate (GTN) group. Comparison by Cox proportional regression. Adjusted HR 0.64 (95% CI 0.40 to 1.02), p=0.062.

In adjusted analyses, the presence of nitrate-related headache was not associated with an improved functional outcome at 90 days: adjusted OR 0.90 (95% CI 0.73 to 1.10; p=0.30) (table 2 and figure 1), or with a better survival up to 90 days: adjusted HR 0.64 (95% CI 0.40 to 1.02; p=0.062) (table 2 and figure 2). However, the presence of a nitrate-related headache by day 7 was associated with a lower risk of death or deterioration at day 7 and death in hospital (table 2). Further, nitrate-related headache was associated with higher/better scores at day 90 for the Barthel Index, MMSE and Telephone Interview of Cognitive Status. A tendency to less early recurrence was also apparent for patients developing a nitrate-related headache (table 2).

**Table 2:** Relationship between outcome and headache by day 7, in participants who received GTN

Headache	Total	Yes	No	Unadjusted OR/HR/MD (95% CI)	2p	Adjusted OR/HR/MD (95% CI)	P value
Day 7 (%)*							
Death	1996	2 (0.6)	59 (3.6)	0.15 (0.04 to 0.61)	0.008	0.24 (0.06 to 1.00)	0.050
Deterioration/death	1992	14 (3.9)	166 (10.2)	0.36 (0.21 to 0.62)	<0.001	0.45 (0.25 to 0.82)	0.009
Recurrence	1995	3 (0.8)	39 (2.4)	0.34 (0.11 to 1.12)	0.076	0.41 (0.12 to 1.36)	0.15
sICH	1996	4 (1.1)	18 (1.1)	1.01 (0.34 to 3.00)	0.99	1.15 (0.36 to 3.69)	0.82
Hypotension	1996	6 (1.7)	50 (3.1)	0.54 (0.23 to 1.26)	0.16	0.52 (0.22 to 1.24)	0.14
Hypertension	1996	40 (11.1)	99 (6.1)	1.94 (1.32 to 2.86)	0.001	1.81 (1.19 to 2.76)	0.006
SSS (/58)	1992	43.4 (13)	38.2 (16.3)	5.25 (3.45 to 7.05)	<0.001	0.88 (−0.25 to 2.01)	0.13
SAEs by day 7 (%)*							
Cerebral oedema	1996	4 (1.1)	11 (0.7)	1.66 (0.53 to 5.24)	0.39	2.90 (0.77 to 10.85)	0.12
Complication	1996	0 (0)	22 (1.3)	–	–	–	–
Extension	1996	5 (1.4)	48 (2.9)	0.47 (0.18 to 1.18)	0.11	0.53 (0.21 to 1.38)	0.19
Hospital events							
Length of stay (days)†	1988	17.8 (20.7)	21.4 (23.9)	−3.6 (−6.0 to −1.1)	0.009	0.4 (−2.1 to 2.9)	0.76
Death (%)‡	1996	10 (2.8)	149 (9.1)	0.29 (0.15 to 0.55)	<0.001	0.44 (0.22 to 0.88)	0.020
Day 90							
Death (%)‡	1991	21 (5.8)	210 (12.9)	0.42 (0.27 to 0.67)	<0.001	0.64 (0.40 to 1.02)	0.062
mRS (/6)§	1996	2.6 (1.6)	3.2 (1.7)	0.58 (0.47 to 0.70)	<0.001	0.90 (0.73 to 1.10)	0.30
Barthel Index (/100)†	1980	77 (31.4)	63 (39.0)	14.0 (10.2 to 17.7)	<0.001	3.7 (0.3 to 7.1)	0.034
MMSE (/30)†	1003	13.8 (6)	10.6 (7.6)	3.2 (2.2 to 4.2)	<0.001	1.5 (0.6 to 2.4)	0.002
TICS-M (/30)†	993	18.5 (8.8)	14 (10.6)	4.5 (3.1 to 5.9)	<0.001	2.0 (0.7 to 3.3)	0.003
Category fluency†	1172	11.6 (7.5)	8.7 (97.7)	2.9 (1.8 to 4.0)	<0.001	1.3 (0.3 to 2.2)	0.008
ZDS (/102.5)†	1630	54.6 (20.5)	59.1 (24.3)	−4.5 (−7.4 to -1.6)	0.002	−0.9 (−3.5 to 1.7)	0.48
EQ-5D/HUS (/1)†	1975	0.52 (0.39)	0.45 (0.4)	0.08 (0.03 to 0.12)	0.001	−0.01 (0.03 to 0.95)	0.69
EQ-VAS (/100)†	1717	60.9 (28.0)	55.5 (31.3)	1.9 (1.6 to 9.1)	0.005	−0.5 (−3.8 to 2.8)	0.76

Adjusted results are shown, with adjustment for age, sex, premorbid mRS score, previous stroke, diabetes mellitus, current nitrate use, time from onset to randomisation systolic blood pressure, total anterior circulation syndrome, Scandinavian Stroke Scale Score, stroke type, thrombolytic treatment, feeding status and continuation of prestroke antihypertensive drugs.

Data are number (%) or mean (SD), and OR, HR or mean difference (95% CIs).

*Comparisons by binary logistic regression.

†Multiple linear regression.

‡Cox regression.

§Ordinal logistic regression.

EQ-5D, European Quality of Life-5 dimensions; EQ-VAS, European Quality of Life-Visual Analogue Scale; HUS, health utility status; ICH, symptomatic intracranial haemorrhage; MD, mean difference; mRS, modified Rankin Scale; SAE, serious adverse event; SSS, Scandinavian Stroke Scale; TICS, Modified Telephone Interview for Cognitive Status; tMMSE, telephone Mini-Mental State Examination; ZDS, Zung Depression Scale.

In patients randomised to no GTN, the presence of a headache by day 7 was not associated with a change in functional outcome at 90 days or with a change in survival, but it was associated with a longer length of stay in hospital, and lower health utility status (as derived from the EQ-5D) at day 90 (online supplemental table 2). Hypertension was significantly associated with both nitrate and non-nitrate headache, but cerebral oedema was only more common in patients with non-nitrate headache (table 2 and online supplemental table 2).

**Figure 3:**
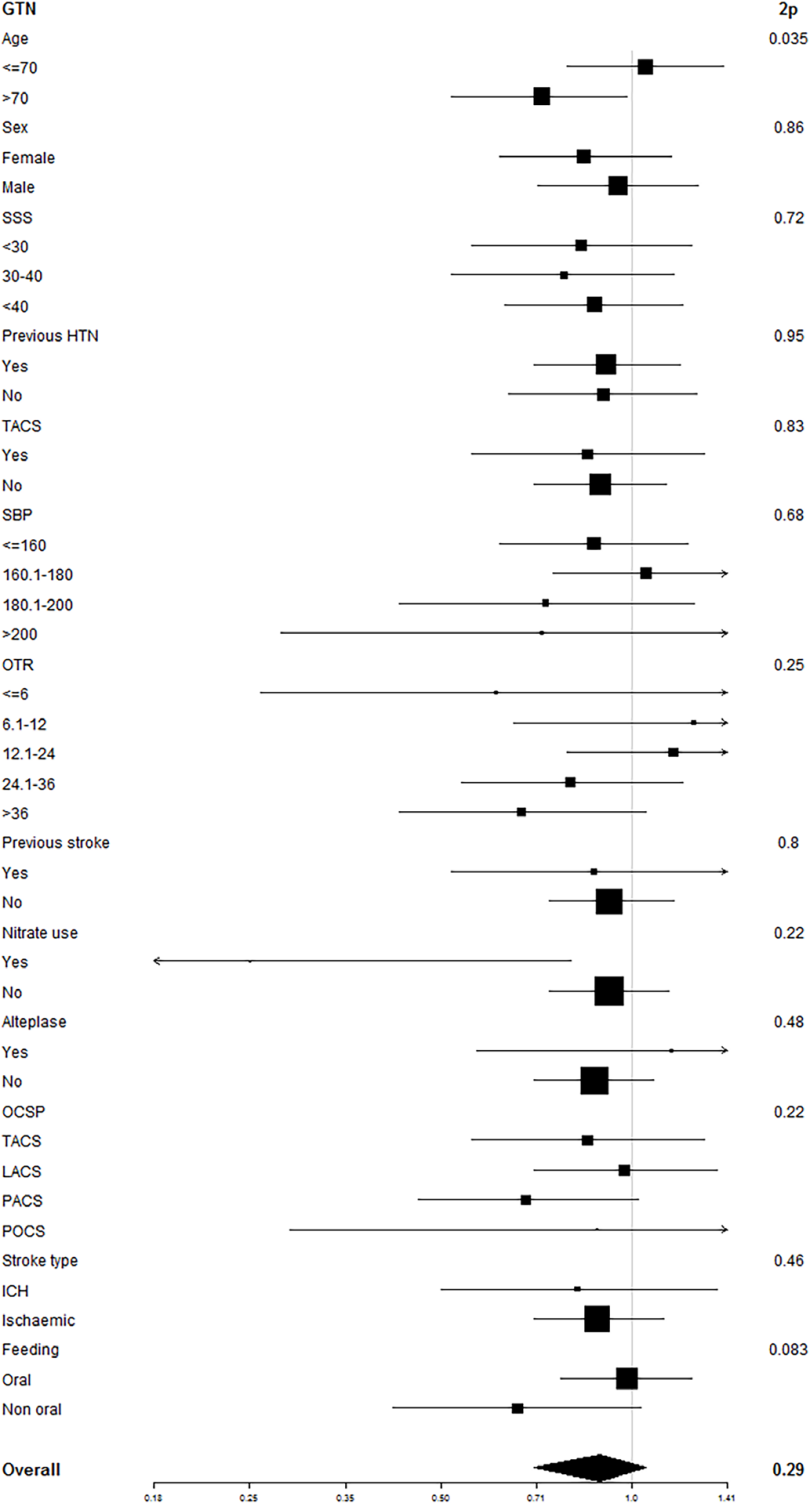
Subgroup analysis for the interaction between baseline parameters and headache on the modified Rankin Scale in patients who received GTN. Data are ORs (95% CI), modelled using ordinal logistic regression with an interaction term and with adjustment (variables listed in statistics section). GTN, glyceryl trinitrate; HTN, hypertension; ICH, intracranial haemorrhage; LACS, lacunar circulation syndrome; OCSP, Oxfordshire Community Stroke Project Classification; OTR, on treatment randomisation; POCS, posterior circulation syndrome; SBP, systolic blood pressure; SSS, Scandinavian Stroke Scale; TACS, total anterior circulation syndrome.

When assessing interactions between headache, baseline factors, and outcome (mRS), participants randomised to GTN were more likely to have a good outcome if they were older (figure 3). In patients who did not receive GTN, a better outcome was apparent in older participants and men who developed a (non-nitrate) headache (online supplemental figure 1).

Finally, in a time-updated analysis, nitrate headache was associated with significantly lower risk of death at between 0 and 7 days after headache onset, but not at other time points, (online supplemental tables 3 and 4). There was no association with non-nitrate headache on survival at different time points, (online supplemental tables 3 and 4). Nitrate headache was associated with increased risk of death, beyond 90 days after headache onset, (online supplemental tables 3 and 4).

## Discussion

This prespecified subgroup analysis of the ENOS trial^18^ reveals that reported headache after stroke and within 7 days is relatively common (8.5%) and that GTN, an organic nitrate that releases nitric oxide, more than doubles this rate to 18.0%. Although development of a nitrate headache within 7 days of randomisation was not associated with improvement in functional outcome or increased survival by 90 days, it was associated with reduced death, and deterioration by day 7, reduced death in hospital, and better activities of daily living and cognitive impairment at day 90. Despite the lack of association between headache and the primary outcome (mRS), activities of daily living as assessed by the Barthel Index, were significantly improved in the nitrate headache group. Although both scales are a measure of disability, the greater focus on specific tasks in the Barthel Index, rather than a global measure of disability provided by the mRS, may be more sensitive to subtle changes, particularly in mild to moderate stroke.^22 23^

The presence of a headache after stroke onset has been associated variously with poor clinical outcome^17^ and reduced risk of recurrence.^12^ In the PRoFESS secondary prevention trial,^11^ development of headache with dipyridamole (a phosphodiesterase inhibitor [PDEI]) was associated with a reduction in stroke recurrence.^12^ A similar result was seen in the ESPS-2 trial.^10^ A tendency to a lower rate of recurrence was also present in our patients with a nitrate headache, although this did not translate into a better functional outcome or survival. Possible reasons for the stronger effect on stroke recurrent in the PRoFESS and ESPS-2 trials may be: longer duration of follow-up, increased number of events (more statistical power), focus on stroke recurrence rather than functional outcome, or the use of alternative nitrates. The development of headache in response to GTN suggests the ability of the cerebral vasculature to dilate,^12^ a mechanism common to migrainous headache.^24^ Dilation of the dysfunctional vasculature may increase collateral vascular efficiency, cerebral blood flow (CBF) and perfusion to viable ischaemic penumbra.^25–27^ Nevertheless, studies of CBF and CBF-velocity did not find, overall, any changes related to administration of GTN.^5 6^

The strengths of this prespecified substudy of the ENOS trial are the large sample size and completeness of data in the ENOS trial, this reducing the risk that the findings are purely due to chance; the international, multicentre setting of the trial which increases the external validity of the findings; and the possibility to adjust in for a number of prospectively collected prognostic variables, especially clinical variables at baseline.

Nevertheless, a number of caveats need highlighting. First, patients who developed a nitrate-headache differed from those who did not have a headache and some of these differences will have favoured outcome in the nitrate-headache group, especially lower age and stroke severity, and lacunar stroke syndrome. Statistical adjustment will have corrected, in part, for some of these differences. Second, the association of nitrate headache with mild stroke (high SSS, non-TACS) may be real but could alternatively reflect that patients with severe stroke or a TACS may have headache but not be able to report it, perhaps due to the presence of dysphasia, confusion or reduced level of consciousness. Similarly, ENOS was patient—but not staff-masked; hence, staff may have been more likely to monitor and report headache in those patients randomised to GTN. Finally, data were not collected on the time to headache development, so it is unclear if headache occurring within 7 days was during the initial or later phase of nitrate treatment. Nitrate headache typically occurs within 6 hours of treatment onset and abates by 5–7 days.^28^ Nitrate headaches occurring within 1 hour of treatment are usually mild to moderate with spontaneous resolution, where-as migraine-type headaches associated with nitrate are delayed (onset 3–6 hours) and are usually more severe and persistent.^28^ Nitrate tolerance (loss of vasodilation) usually occurs within 24–48 hours of treatment, and it remains unclear why headache persists after this time.^28^ Further work, exploring the time to headache onset could identify whether earlier or later onset nitrate-headaches are associated with improved outcomes after stroke.

In conclusion, the development of nitrate-related headache at day 7 after acute stroke may have important prognostic implications and was associated with reduced death, deterioration at day 7, and improved activities of daily living and cognition scores at 90 days. These findings will be reassessed in the ongoing rapid implementation of GTN in hypertensive stroke trial-2.

## Data Availability

Data are available on reasonable request. Individual participant data will be shared with the Virtual International Stroke Trials Archive (VISTA) collaboration. From 1 January 2022, the chief investigator and trial steering committee will consider other requests to share individual participant data via email at: enos@nottingham.ac.uk. We will require a protocol detailing hypothesis, aims, analyses and intended tables and figures. Where possible, we will perform the analyses; if not, deidentified data and a data dictionary will be supplied for the necessary variables for remote analysis. Any sharing will be subject to a signed data access agreement. Ultimately, the data will be published.
