# Supplementary material for: Relationship between nitrate headache and outcome in patients with acute stroke: results from the efficacy of nitric oxide in stroke (ENOS) trial
Source: Stroke Vasc Neurol. 2020 Nov 5;6(2):180–6. doi: 10.1136/svn-2020-000498 (PMC8258084; doi:10.1136/svn-2020-000498)

SUPPLEMENTARY INFORMATION

Contents

	Heading	Page
Tables		
1	Change in haemodynamic measures from baseline to day 1 by treatment group and development of headache	2
2	Relationship between outcome and headache by day 7, in participants who did not receive GTN	3
3	Relationship between headache and death in patients who received GTN, using time updated co-variates	4
4	Relationship between headache and death in patients who did not receive GTN, using time updated co-variates	5
Figure		
1	Distribution of modified Rankin Scale scores at day 90 by presence or absence of headache by day 7 for the no GTN group	6
2	Survival curves over 90 days of follow up for no GTN treatment group.	7
3	Sub-group analysis for the interaction between baseline parameters and headache on the modified Rankin Scale in patients who did not receive GTN.	8

Supplementary Table 1. Change in haemodynamic measures from baseline to day 1 by treatment group and development of headache.
Data are change from baseline (standard deviation), mean difference (95% confidence interval).

	GTN				No GTN			
	Headache	No headache	MD (95% CI)	P value	Headache	No headache	MD (95% CI)	P value
SBP (mmHg)	-9.9 (16.92)	-10.7 (19.6)	0.85 (-1.37, 3.07)	0.41	-2.1 (19.3)	-3.7 (18.3)	1.56 (-1.36, 4.48)	0.30
DBP (mmHg)	-5.8 (11.92)	-5.1 (12.5)	-0.62 (-2.06, 0.82)	0.40	-0.6 (11.1)	-1.7 (11.7)	1.06 (-0.79, 2.91)	0.26
Heart rate (bpm)	1.4 (10.49)	1.2 (10.6)	0.18 (-1.05, 1.41)	0.77	1.1 (8.9)	-0.1 (10.0)	1.21 (-0.36, 2.79)	0.13
PP (mmHg)	-4.1 (13.69)	-5.6 (14.8)	1.47 (-0.22, 3.16)	0.089	-1.5 (14.4)	-2.0 (14.7)	0.50 (-1.84, 2.84)	0.68
MAP (mmHg)	-7.1 (12.18)	-7.0 (13.6)	-0.13 (-1.67, 1.41)	0.87	-1.1 (12.7)	-2.3 (12.4)	1.22 (-0.75, 3.2)	0.23
RPP (mmHg.bpm)	-554 (2206)	-611 (2418)	56.6 (-219.6, 332.8)	0.69	38.6 (2232)	-296 (2260)	335.0 (-25.5, 695.4)	0.069

bpm: beats per minute; CI: confidence intervals; DBP: diastolic blood pressure; MAP: mean arterial pressure; MD: mean difference; PP: pulse pressure; RPP: rate pressure product (= systolic BP x heart rate); SBP: systolic blood pressure

Supplementary Table 2. Relationship between outcome and headache by day 7, in participants who did not receive GTN.

Data are number (%) or mean (standard deviation), and odds ratio, hazard ratio or mean difference (95% confidence intervals). Comparisons by binary logistic regression[1], multiple linear regression[2], Cox regression[3], or ordinal logistic regression[4]. Unadjusted and adjusted results are shown, with adjustment for age, sex, pre-morbid modified Rankin scale, previous stroke, diabetes mellitus, current nitrate use, time from onset to randomisation systolic blood pressure, total anterior circulation syndrome, Scandinavian Stroke Scale, stroke type, thrombolysis given, feeding status, and randomisation to continue versus stop pre-stroke antihypertensive drugs.

Headache	Total	Yes	No	Unadjusted OR/HR/MD (95% CI)	2p	Adjusted OR/HR/MD (95% CI)	2p
Day 7 (%)[1]							
Death	2004	3 (1.8)	55 (3)	0.58 (0.18, 1.88)	0.36	0.75 (0.22, 2.51)	0.64
Deterioration/death	1999	12 (7.1)	186 (10.2)	0.67 (0.37, 1.23)	0.2	0.65 (0.35, 1.23)	0.19
Recurrence	2002	3 (1.8)	28 (1.5)	1.16 (0.35, 3.85)	0.81	1.24 (0.36, 4.26)	0.73
sICH	2004	1 (0.6)	17 (0.9)	0.63 (0.08, 4.78)	0.66	0.7 (0.09, 5.61)	0.74
Hypotension	2004	0 (0)	16 (0.9)	-	-	-	-
Hypertension	2004	25 (14.7)	132 (7.2)	2.22 (1.4, 3.52)	0.001	1.96 (1.21, 3.19)	0.006
SSS (/58)	1999	39.6 (15.3)	38.4 (16.4)	1.3 (-1.3, 3.8)	0.33	0.8 (-0.7, 2.3)	0.31
SAEs by day 7 (%)[1]							
Cerebral oedema	2004	4 (2.4)	15 (0.8)	2.92 (0.96, 8.91)	0.06	4.94 (1.36, 17.94)	0.015
Complication	2004	0 (0)	11 (0.6)	-	-	-	-
Extension	2004	1 (0.6)	32 (1.7)	0.33 (0.05, 2.45)	0.28	0.37 (0.05, 2.78)	0.34
Hospital events							
Length of stay (days)[2]	1997	25.1 (27.5)	20.7 (23.4)	4.4 (0.1, 8.7)	0.020	5.5 (2.0, 9.0)	0.002
Death (%)[3]	2004	10 (5.9)	151 (8.2)	0.7 (0.36, 1.34)	0.28	0.96 (0.47, 1.98)	0.92
Day 90							
Death (%)[3]	1995	17 (10.1)	246 (13.5)	0.72 (0.43, 1.21)	0.21	0.93 (0.56, 1.53)	0.77
mRS (/6)[4]	2004	3.12 (1.6)	3.11 (1.8)	1.03 (0.78, 1.36)	0.85	1.15 (0.89, 1.4)	0.35
Barthel Index (/100)[2]	1982	65.6 (35.9)	63.1 (39.7)	2.53 (-3.23, 8.29)	0.43	-2.4 (-5.00, 4.52)	0.92
MMSE (/30)[2]	1021	12.2 (7)	10.7 (7.8)	0.8 (0.00, 3.1)	0.066	0.4 (-0.9, 1.6)	0.57
TICS-M (/30)[2]	1013	15.8 (9.5)	14.4 (10.9)	1.5 (-0.7, 3.6)	0.22	-0.2 (-2.0, 1.6)	0.81
Category fluency[2]	1185	10.2 96.9)	9.1 (8)	1.1 (-0.2, 2.5)	0.14	0.2 (-1.1, 1.5)	0.77
ZDS (/102.5)[2]	1615	58.2 (23.4)	58.9 (24.7)	-0.7 (-5.0, 3.7)	0.76	1.0 (-2.7, 4.7)	0.58

EQ-5D / HUS (/1)[2]	1968	0.42 (0.4)	0.46 (0.4)	-0.04 (-0.10, 0.03)	0.24	-0.06 (-0.11, 0.01)	0.024
EQ-VAS (/100)[2]	1715	57.7 (30.6)	55.4 (31.7)	2.8 (-3.1, 7.7)	0.41	-0.4 (-5.00, 4.3)	0.88

EQ-5D: European Quality of Life-5 dimensions; EQ-VAS: European Quality of Life-Visual Analogue Scale; HUS: health utility status; mRS: modified Rankin Scale; sICH: symptomatic intracranial haemorrhage; SSS: Scandinavian Stroke Scale; tMMSE: telephone Mini Mental State Examination; TICS: Modified Telephone Interview for Cognitive Status; ZDS: Zung Depression Scale.

Supplementary Table 3. Relationship between headache and death in patients who received GTN, using time updated co-variates (days 0-7, days 8-30, day 31-90 to death after headache occurrence).

Data are number (%), and odds ratio. Comparisons by binary logistic regression. Unadjusted and adjusted results are shown, with adjustment for age, sex, pre-morbid modified Rankin scale, previous stroke, diabetes mellitus, current nitrate use, time from onset to randomisation systolic blood pressure, total anterior circulation syndrome, Scandinavian Stroke Scale, stroke type, thrombolysis given, feeding status, and randomisation to continue versus stop pre-stroke antihypertensive drugs.

Headache	Total	Yes	No	Unadjusted OR/HR/MD (95% CI)	2p	Adjusted OR/HR/MD (95% CI)	2p
Death (%)							
Day 0-7	442	2 (3.8)	66 (17)	0.19 (0.05, 0.81)	0.024	0.21 (0.05, 0.94)	0.042
Day 8-30	441	80	361	0.91 (0.43, 1.96)	0.82	0.78 (0.34, 1.79)	0.56
Day 31-90	441	56	385	0.85 (0.34, 2.08)	0.72	0.88 (0.32, 2.43)	0.8
Day 90+	441	206	236	2 (1.09, 3.68)	0.026	2.38 (1.17, 4.83)	0.017

Supplementary Table 4. Relationship between headache and death in patients who did not receive GTN, using time updated co-variates (days 0-7, days 8-30, and days 31-90 death after headache occurrence).
Data are number (%) and odds ratio. Comparisons by binary logistic regression. Unadjusted and adjusted results are shown, with adjustment for age, sex, pre-morbid modified Rankin scale, previous stroke, diabetes mellitus, current nitrate use, time from onset to randomisation systolic blood pressure, total anterior circulation syndrome, Scandinavian Stroke Scale, stroke type, thrombolysis given, feeding status, and randomisation to continue versus stop pre-stroke antihypertensive drugs.

Headache	Total	Yes	No	Unadjusted OR/HR/MD (95% CI)	2p	Adjusted OR/HR/MD (95% CI)	2p
Death (%)							
Day 7	442	2 (5.3)	59 (13.8)	0.35 (0.08, 1.47)	0.15	0.24 (0.05, 1.12)	0.070
Day 8-30	462	96	366	1.02 (0.45, 2.3)	0.97	1.04 (0.43, 2.48)	0.93
Day 31-90	462	72	390	0.8 (0.3, 2.12)	0.65	0.93 (0.33, 2.6)	0.89
Day 90+	464	232	232	1.59 (0.81, 3.14)	0.18	1.72 (0.8, 3.71)	0.17

Supplementary Figure 1. Distribution of modified Rankin Scale scores at day 90 by presence or absence of headache by day 7 for the no GTN group.
Data are percentage of total patients. Comparison by ordinal logistic regression.

No GTN group: Adjusted odds ratio 1.15 (0.89, 1.4), 2p=0.35 (No headache: n=1826, headache: n=169).

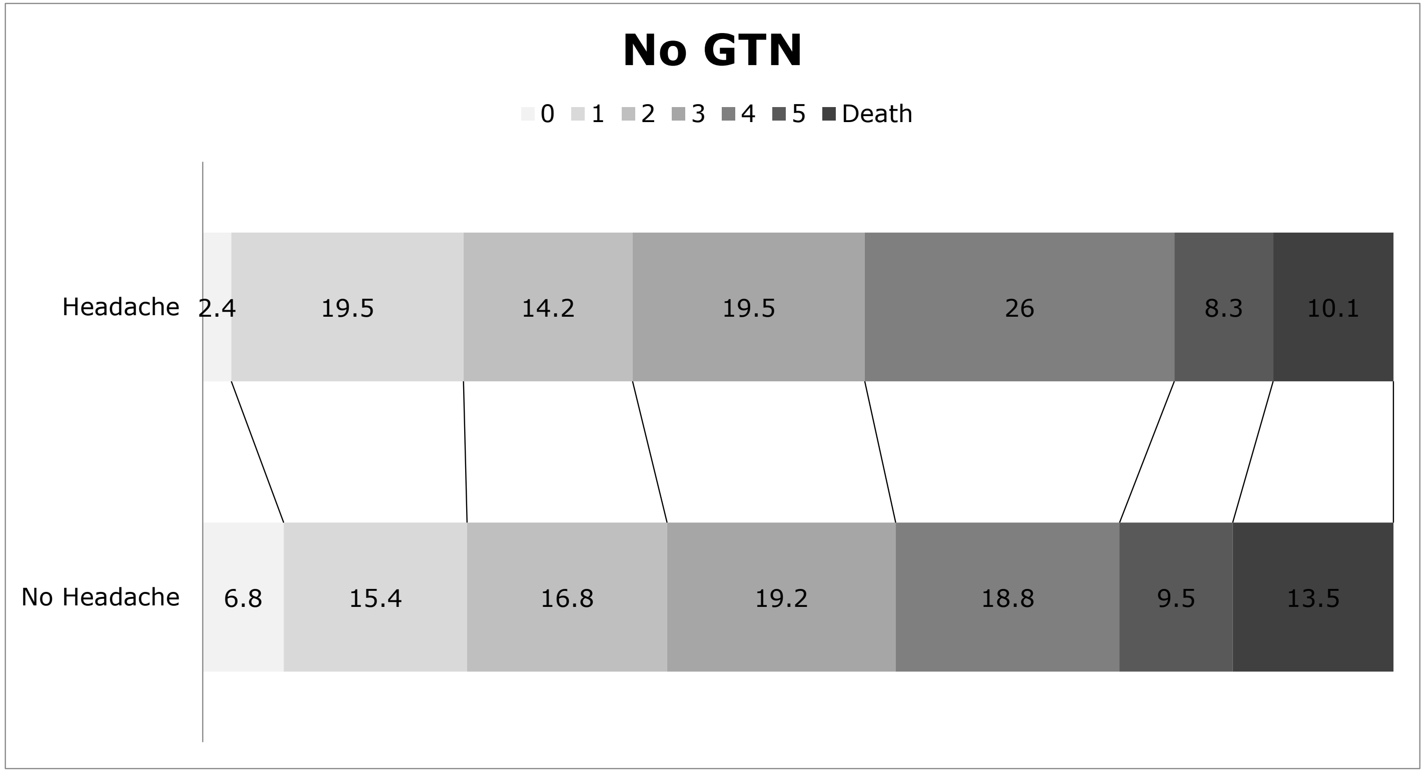

Supplementary Figure 2. Survival curves over 90 days of follow up for no GTN treatment group.
Comparison by Cox proportional regression.

Adjusted hazard ratio 0.93 (0.56, 1.53), p=0.77.

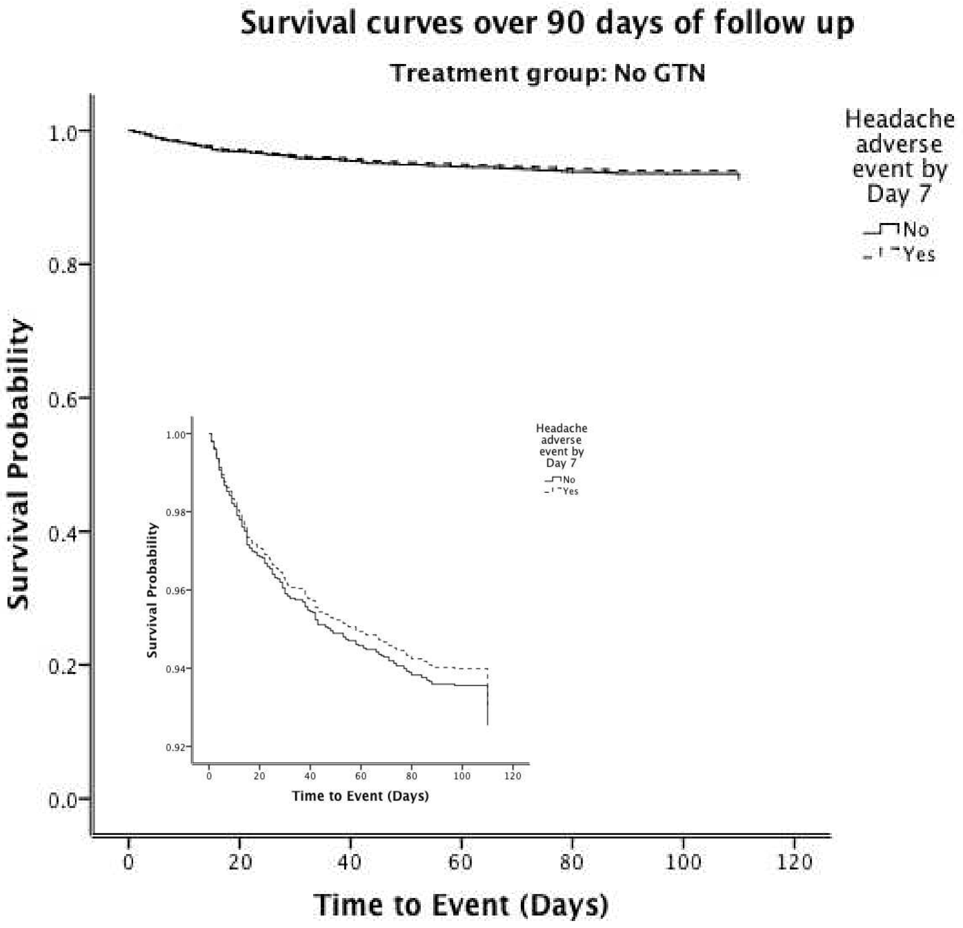

Supplementary Figure 3. Sub-group analysis for the interaction between baseline parameters and headache on the modified Rankin Scale in patients who did not receive GTN.
Data are odds ratio (95% confidence interval), modelled using ordinal logistic regression with an interaction term and with adjustment (variables listed in statistics section). HTN: hypertension; OTR: on treatment randomisation; SBP: systolic blood pressure; SSS: Scandinavian Stroke Scale; TACS: total anterior circulation syndrome.

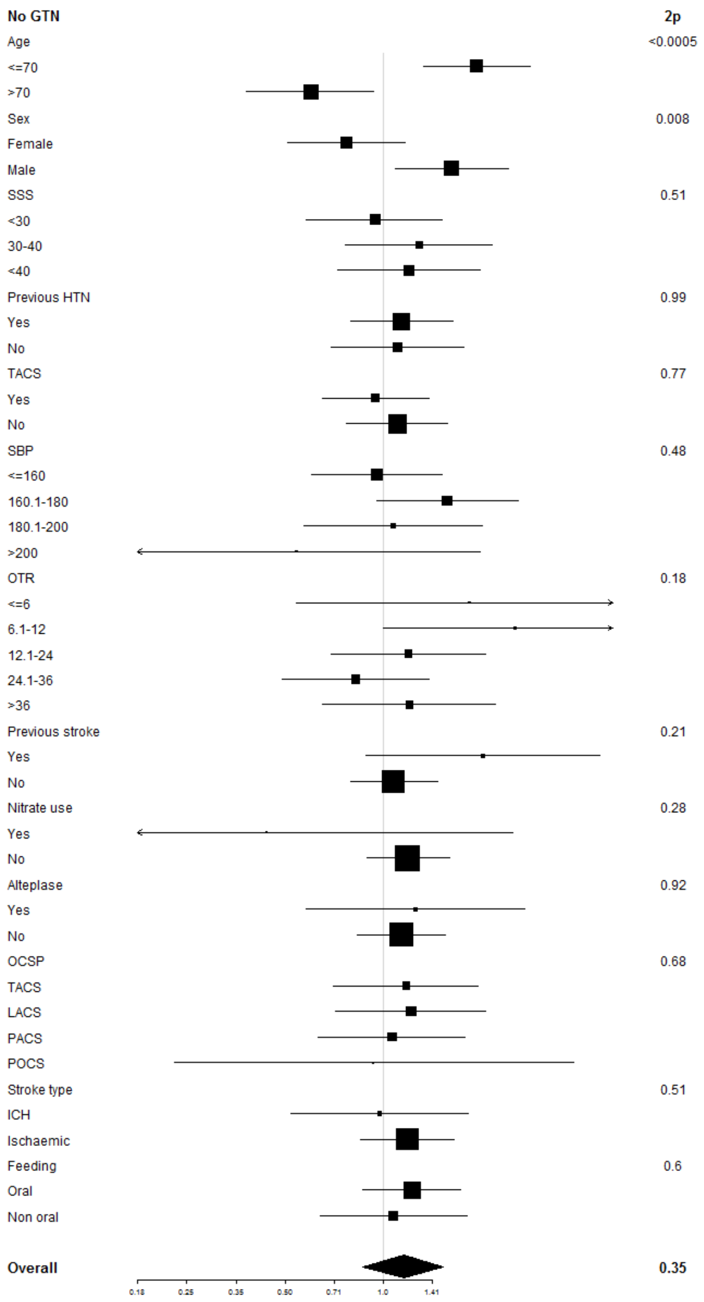

Supplement: Supplementary data [file svn-2020-000498supp001.pdf]
